# Flipping the Thinking on Equality, Diversity, and Inclusion. Why EDI Is Essential for the Development and Progression of the Chemical Sciences: A Case Study Approach

**DOI:** 10.1021/acs.jchemed.3c00364

**Published:** 2023-10-12

**Authors:** M. Anwar H. Khan, Timothy G. Harrison, Magdalena Wajrak, Michele Grimshaw, Kathy G. Schofield, Alison J. Trew, Kulvinder Johal, Jeannette Morgan, Karen. L. Shallcross, Joyce D. Sewry, Michael T. Davies-Coleman, Dudley E. Shallcross

**Affiliations:** † School of Chemistry, Cantock’s Close, University of Bristol, Bristol BS8 1TS, United Kingdom; ‡ School of Science, 270 Joondalup Drive, Edith Cowan University, Perth, WA 6027, Australia; § Primary Science Teaching Trust, 12 Whiteladies Road, Bristol BS8 1PD, United Kingdom; ∥ Department of Chemistry, Rhodes University, Makhanda 6139, South Africa; ⊥ Department of Chemistry, Robert Sobukwe Drive, University of Western Cape, Bellville 7535, South Africa

**Keywords:** Elementary/Middle School Science, High School, First-Year Undergraduate, Second-Year Undergraduate, Upper-Division Undergraduate, Graduate Education, Curriculum/Outreach, Analogies/Transfer, Inclusion

## Abstract

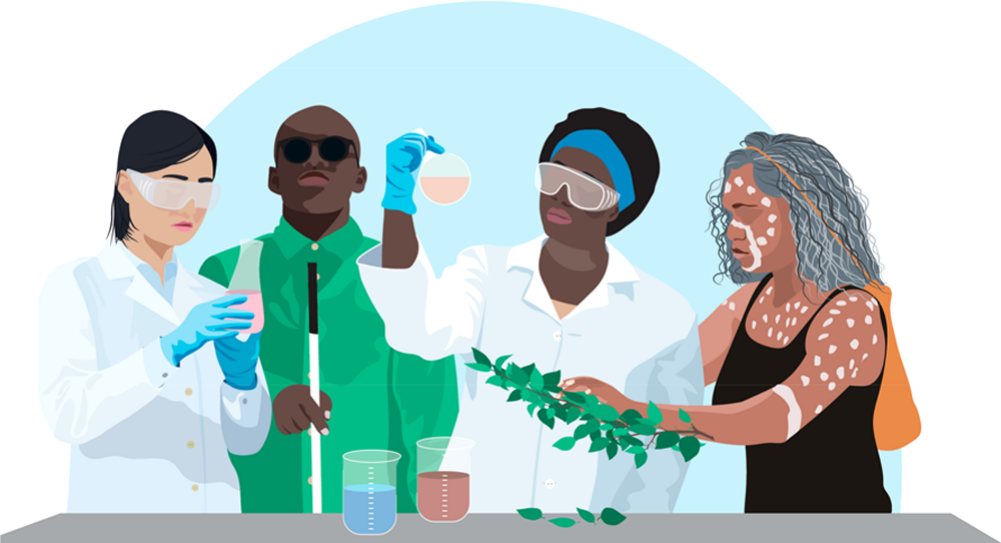

All learners have a contribution to make to the development of the Chemical Sciences, be that in novel ways to teach, and their perspectives and contexts, but also in research, both in chemical education and the wider Chemical Sciences. Through four case studies, this paper explores interactions with diverse groups and how this has altered perspectives on both teaching and research. The case studies include work with visually impaired adults, a project bringing together First Peoples in Australia with academics to explore old ways (traditional science) and new ways (modern approaches), primary (elementary) school perspectives on teaching science, and a project in South Africa to connect university and township communities. Not only do these case studies demonstrate the immense value these diverse groups bring to our understanding about how to learn, but they also bring new perspectives on how to view and solve chemical problems.

## Introduction

Here, defining EDI as equality, diversity, and inclusion, we note that EDI is, quite rightly, becoming prominent elements of chemical science undergraduate and postgraduate courses. Defining disability, for example, is difficult,^1^ with a wide range of areas to consider, but “disability” is not the only issue to address in the Chemical Sciences. The chemical laboratory is an important component of any degree^2^ but is typically not inclusive and accessible.^3^ Physical impairments such as hearing loss^4^ and visual impairment^5−8^ are challenging, but solutions can be envisaged and enacted; however, more subtle challenges such as neurodiversity^9^ are far more difficult to identify and resolve. It is noted that the laboratory is an environment that can overload the senses for both learner and instructor.^10^ Various adaptations can be made that would benefit numerous learners such as physical adjustments, and accessible resources such as apps can be effective,^11^ but what can the educator learn from interacting with a much wider cohort of learners?

Recognizing and supporting learners concerning EDI, e.g., through (compulsory) training^12−14^ or discussion events,^15^ can indeed foster an inclusive culture, but can we then incorporate elements of excellent practice from cultures that are not represented as well as adjust what is already offered? All efforts to identify and address issues concerning EDI and the teaching of the Chemical Sciences are essential. Indeed, in primary schools in the U.K., where ethnic groups may represent the majority of the school population, using other cultures as a context for learning is not only desirable but also can lead to excellent opportunities to explore different contexts, e.g., the way you make tea in different cultures.^16^ Therefore, educators who work with a very different cultural mix have much experience that can be shared. However, apart from the desire to level the playing field and provide equal opportunities to all, there is a more fundamental reason: these learners provide insights to cognition that can enhance the learning of all. For example, in the second case study we consider a partnership with First Peoples in Australia and how they devised ways to clean themselves and fabricate a nappy well before soap and polymer-based nappy linings were invented. Learning about their experimental methods, their use of natural and readily available resources, and the way that knowledge is passed on to next generations provides a different and highly valuable dialogue about the chemical method.

In this paper, we look at four case studies that demonstrate some of the many and varied lessons that can be learned to improve teaching for all by engaging with a diverse group of learners and practitioners. Ultimately, the Chemical Sciences will advance in teaching if it engages with all learners, but research too will be more diverse in thinking, and one assumes this will have myriad benefits to advancements in the subject. Decolonizing the curriculum,^12,17−20^ for example, is an important area that we address in more detail in case 2. In a desire to have a global perspective on chemistry (science) and its development, we need to hear global voices.^19^ The design of curricula, the content, contexts, and the way ideas are progressed are often embedded in a western and northern hemispheric perspective. The purpose of decolonization, in part, is to challenge and change this perspective, so that a better product for all learners is produced.

Through the Bristol ChemLabS outreach program (housed by the School of Chemistry at the University of Bristol, U.K.),^21,22^ which has run for ca. 18 years, a variety of projects has sought to address EDI in the Chemical Sciences and has involved partners from Australia and South Africa (reported here) but also many other countries. We draw on four case studies from the Bristol ChemLabS Outreach program and its partners that illustrate benefits of embracing EDI for future students and current and future educators. A description of the work carried out in each case study is presented in the Supporting Information. The four case studies consider the following: working with visually impaired adults, working with First Peoples in Australia, working with primary (elementary) school children in the U.K., and working with local communities housed in townships in South Africa.

## Case Studies

### Case Study 1: Visually Impaired Adults^23^

The opportunities for groups with disabilities to engage with chemistry and in particular practical chemistry are all too rare. Where this has taken place, it has involved, for example, web-based reading,^24,25^ the use of QR codes to facilitate knowledge transfer via the spoken word,^26^ computational chemistry via talking computers,^27,28^ a summer school for preuniversity students,^29^ and an undergraduate course where scribes and other guides were used to support a legally blind undergraduate.^30^ Health and safety considerations^31,32^ are often barriers to prevent such interactions. However, over the course of three years, a summer school for a cohort of visually impaired adults was run by the Science Faculty at the University of Bristol, U.K. (see the supplementary document for more details). It turns out that if thorough risk analyses are undertaken and of course elevated health and safety considerations, safe and insightful practical investigations can take place, even with guide dogs present. Health and safety considerations were required for the dogs, e.g., reducing the chance of loud bangs. It emerges that sighted guides with limited science knowledge can present a more significant safety hazard (if not properly trained in laboratory safety) than the visually impaired participants. Therefore, experienced demonstrators (postgraduate students with at least two years’ experience in demonstrating to undergraduate chemists) are much safer and can learn rapidly how to work with visually impaired adults. There is no doubt that the experience for the visually impaired adults was highly valued, with much positive feedback. However, the impact on all scientists who took part in the summer schools was invaluable and changed approaches to teaching familiar subjects and prompted different thinking on research as will be described.

Academics from the Chemical Sciences rarely encounter students with visual impairment and, as a result, rarely consider teaching approaches that are not dominated by visual interaction. Take away the ability to see, and one must think about engaging other senses. Physical models that can be touched,^5^ chemicals that can be smelled or tasted, engaging an audience with your voice, and employing good story-telling techniques^33^ are all important. Several teachers of the course reported that they were going to revamp courses they had given for many years, excited by the opportunities offered by working with this cohort. In the new degree course at the School of Chemistry, there are 2 h undergraduate workshops instead of 1 h, with a change of emphasis on exploring the topic using simulations, physical models where appropriate, and group challenges, rather than working through questions. Changes were directly influenced by these summer schools. Academics involved with the summer school have changed their approach to lectures (where flipping was not viable) with an emphasis on interactive activities (e.g., demonstrating an experiment). One academic revamped their course so that readily available resources could be used to carry out “kitchen chemistry” experiments in preparation for workshops before we experienced COVID. During COVID, such activities were more prominent and shown to be highly effective.^34^

The pace of interaction with such groups is naturally slower; they need time to engage with samples or activities, and this is an important consideration in traditionally tightly timetabled teaching sessions. The groups encountered were clearly enthusiastic and wanted to be there, and they asked a lot of questions and were keen to take the time to discuss the topics raised with much peer-to-peer discussion and support.^35^ Such peer-to-peer interactions were extremely powerful and prompted teachers to think about ways that this could be encouraged in their own university workshop sessions. It is hard to stimulate discussion with workshop groups in undergraduate courses, where students are focused on gathering information on how to prepare for the course assessment. In the new course in the School of Chemistry, assessment is no longer 100% by final exam but includes 50% coursework which includes some work in workshops. When discussing molecular structure using stick and ball models, the visually impaired adults made a small segment of silica, and when told that this formed a macromolecule, they all naturally connected their segments together and explored this larger structure. An unplanned session on the properties of this macromolecule occurred. We have run similar workshops with post-16 students and foundation level students (18+) and have never seen them interact spontaneously in this way. In part, the structure of the teaching session with sighted students was short in time (1 h) with a focus on answering some written questions. Since the new course was introduced, as stated, workshop time has been increased. Although most groups require prompting to start to build a macromolecule from base units, we have witnessed some groups repeat the unsighted student’s activity. We believe that the emphasis on hands-on model construction, the time to develop ideas, and a change in assessment focus, all prompted (apart from the assessment method for the nonsighted course) by these workshops, has altered the mode of teaching and has seen a different and positive outcome. Flexibility and being prepared to hand over leading of teaching to the student are important considerations for any student group, but these workshops reminded the academics that students are receptive when teaching sessions are well constructed.

**Figure 1:**
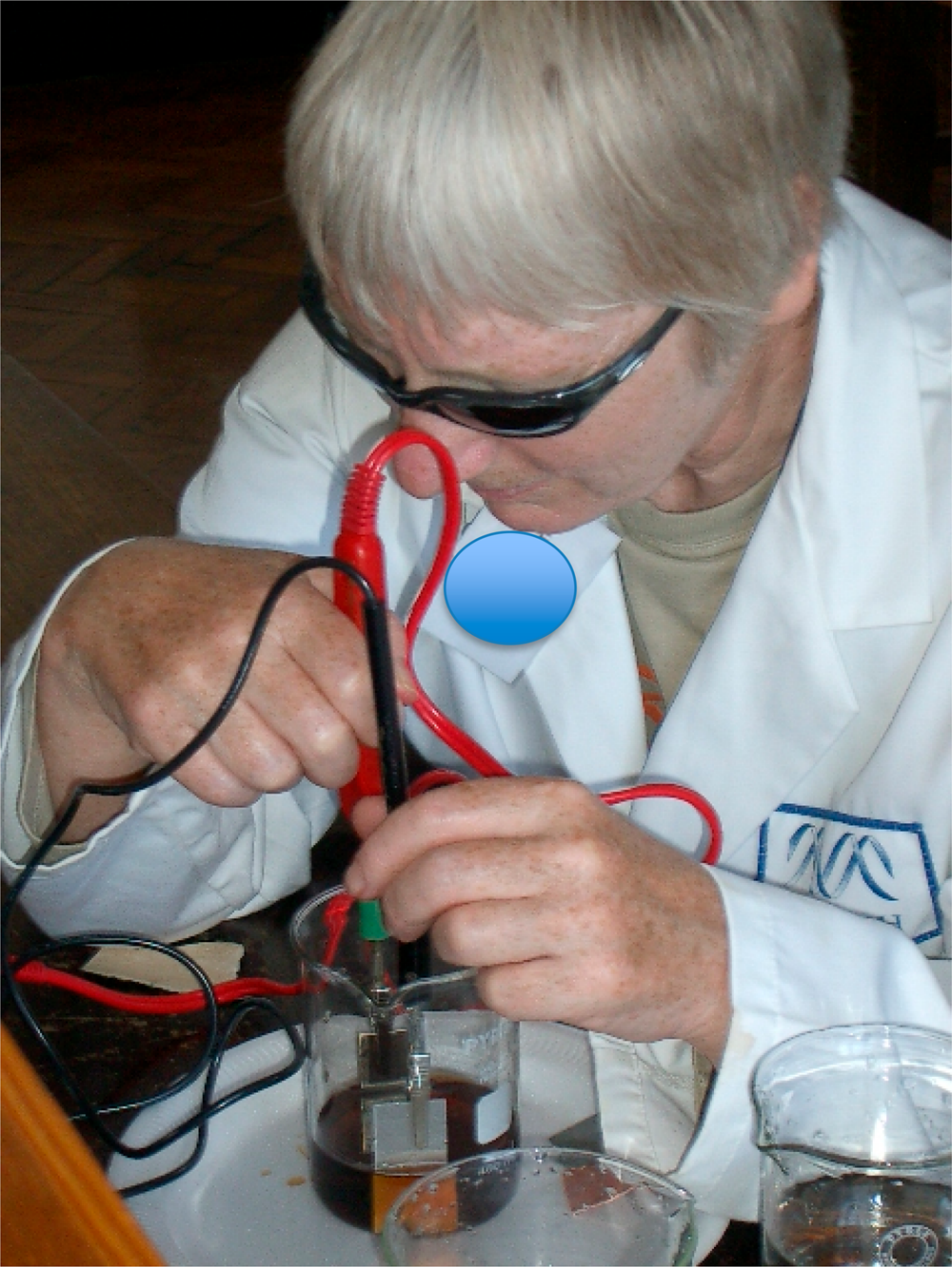
A visually impaired adult carrying out an electrochemistry experiment using a talking voltmeter.

However, undertaking practical investigations was the most challenging but also the most insightful. The use of talking instruments^36,37^ (see Figure 1) was innovative, something that has been used in practicals in undergraduate programs, and aided experimental work with this cohort, and prelab preparation was essential. The setting up of braille-labeled equipment and the time for the students to investigate the pieces via touch were considered. Experiments were designed to be fun, e.g., making the perfect chocolate mixture that melted in your mouth and not in your hand, and the tenacity of the visually impaired adults was impressive as they painstakingly experimented with different mixtures of ingredients. The most telling insight was where they carried out experiments by instructing a demonstrator. There are many learners who struggle to undertake practical work for many reasons other than visual impairment but could benefit considerably from working with a demonstrator. The visually impaired adults worked so effectively with their experienced sighted demonstrators that the demonstrators commented that their approach to demonstrating in general would change. Such changes by demonstrators include having a preliminary discussion with students before laboratory sessions start to ascertain if they have any particular support needs or concerns about the practical, something we assumed that demonstrators did do already but have added to their training. Having worked with nonsighted students, these demonstrators state that their confidence in being able to support undergraduates in the laboratory has increased. It is hard to imagine how these changes in attitude to teaching and demonstrating could have arisen without engaging with this cohort.^38^

An example of these interactions aiding research was when considering air pollution, how it arises, what the health impacts will be and what the current research tells us. Students in the course remarked that they could smell some pollutants, especially those from nascent vehicle emissions, and of course they used sound as an indicator of or proxy for air pollution and used both senses to reduce their exposure. Developing this idea has led to a project that uses a sound sensor in concert with air pollution sensors and has helped primary schools to develop clean walking routes to school and the idea of city soundscapes providing additional information on pollution levels and nature-driven sounds.^39^ Interacting with this cohort has changed teaching practice for the good, it has allowed students who would experience restrictions to benefit from, and contribute significantly to, the development of laboratory teaching and, in several examples, contribute to the development of research.

### Case Study 2: First Peoples in Australia

In 2013 the “Old Ways New Ways” (OWNW) project was developed by a chemistry academic and a Cultural Awareness Officer at Edith Cowan University (ECU) in Western Australia. The focus of the program is to inspire, engage, and empower Aboriginal and Torres Strait Islander students in sciences. The program brings together Western and Indigenous knowledge perspectives in science. Through peer-supported learning and demonstrator training, the program seeks to enhance confidence, leadership, and communication skills for teachers and students. The program introduces and further develops positive role models that inspire the students to consider and explore education and potential science career opportunities. A brief accessible introduction to the program can be found at https://www.ase.org.uk/system/files/Old%20Ways%2C%20New%20Ways.pdf, following presentations of the program at the 2019 Primary Science Education Conference at Edinburgh U.K., hosted by the Primary Science Teaching Trust.

The OWNW program is closely linked to a “dual lens” approach theme of teaching particularly in Indigenous communities. This approach was first developed by an Australian Aboriginal man Pincher Nyurrmiyarri in 1976; it was called “Two Ways” and was a way of “*developing the primary and secondary educational setting to re-establish learning/teaching relationships between old and young and healing rifts in the transmission of traditional knowledge through the interference of schools*” (taken from Ober and Bat, 2007, p 70).^40^ This “Two Ways” approach was further developed and defined by staff at Batchelor Institute as a “*philosophy of education that brings together Indigenous Australian traditions of knowledge and Western academic disciplinary positions and cultural context, and embraces values of respect, tolerance and diversity* (taken from the Bachelor Institute, 2007, p 4).^41^ It has been noted that a “Two Way” curriculum would assist Aboriginal students in mainstream educational systems.^41^ Hence, the motivation for the OWNW program run by Edith Cowan University was clear, improving participation of Aboriginal and/or Torres Strait Islander students in science subjects at school and higher education (HE). In this way, these groups see enhanced employment prospects in science and technology careers. The program delivers hands-on science activities incorporating traditional Aboriginal tool making and ancient techniques for bush survival and sustainability linked to practical experiments in forensic chemistry, such as fingerprinting (see the supplementary document for a list of activities). The workshops are adapted to the differing requirements of students’ age, cognition, and literacy levels (see https://www.ase.org.uk/system/files/Old%20Ways%2C%20New%20Ways.pdf). The implementation of OWNW has been widespread with over 3000 primary and high school students and over 100 teachers across the state, having taken part between 2013 and 2018.

The OWNW program allows young people to see value in the rich heritage of Australia’s First Peoples and the significance of their knowledge to contemporary Australians. The celebration and showcasing of traditional aspects of science by Nyoongar Elders reinforces cross-cultural collaboration and increases respect for traditional knowledge and perspectives. This partnership approaches values and embeds science and the different, yet equally valid, approaches taken by the partners. It has proved to be a most effective way to encourage the target group to view science in a more favorable light, to raise not only their aspirations but also attainment.

A recurring theme in these case studies is time; during these workshops there was a slower reflective pace to them. In a tool-making session using resin, charcoal, and kangaroo faeces (“noongar”), an excellent glue was created, contrasted with the interesting properties of the polymer “polymorph”, whose properties change with temperature, just like the traditional mixture to make the Aboriginal glue. Thinking about how the Aboriginals developed this and the scientific process they went through without formally recognizing it as such was insightful. Working with the environment they inhabit, understanding the natural rhythms and changes, and having an encyclopedic knowledge of the flora and fauna, often through an oral rather than written tradition, were inspiring. We do use and seek biomimetics, but in many environments we have little or no idea about the environment we inhabit and its potential to aid solutions to scientific problems that we have. Once again it was fascinating to work with people who have a heightened sense of smell who use specific chemical odors in the air to identify animals approaching. However, the most fascinating component was the use of the Zamia plant as a nappy. Although the natural material is not able to soak up as much water as the special types of hydrogel polymer used in modern nappies, the environmental aspects of this material and the research process that went into using this material were impressive.

The program team comprises both Aboriginal and non-Aboriginal staff, which models strong cross-cultural partnerships. There is a strong partnership between ECU’s School of Science and its Centre for Aboriginal and Torres Strait Islander Education and Research: Kurongkurl Katitjin. Kurongkurl Katitjin is a Nyoongar phrase meaning “coming together to learn” (see https://www.ase.org.uk/system/files/Old%20Ways%2C%20New%20Ways.pdf, for example, for further information). Whether in teaching or research-led activities, the mind set of Kurongkurl Katitjin, where everyone’s contributions are valued and time and space are made to interact, was a very powerful one. On learning about this approach, a U.K. teacher asked whether there were packs available to create the glue, and the Aboriginal instructor smiled and said that part of the journey is to gather these for yourself. The instructors were not being awkward but were making an important point that in our approach to teaching we often try to set up a path that is clear (to the instructor) but rigid through the material to be covered, and we expect students to grasp ideas and concepts and move on. However, the students have not invested time, made mistakes, become frustrated, and wrestled with the problem to tease out the key contexts and ideas they need. Sometimes, it is important for students to go on a journey where they find out for themselves; they will learn much more and develop a deeper understanding of the subject and associated material. Our own intended learning objectives can blunt learning. The emerging concept of decolonizing^17−20^ the curriculum was addressed directly and very effectively for all parties.

### Case Study 3: Primary (Elementary) School Science Teachers

**Figure 2:**
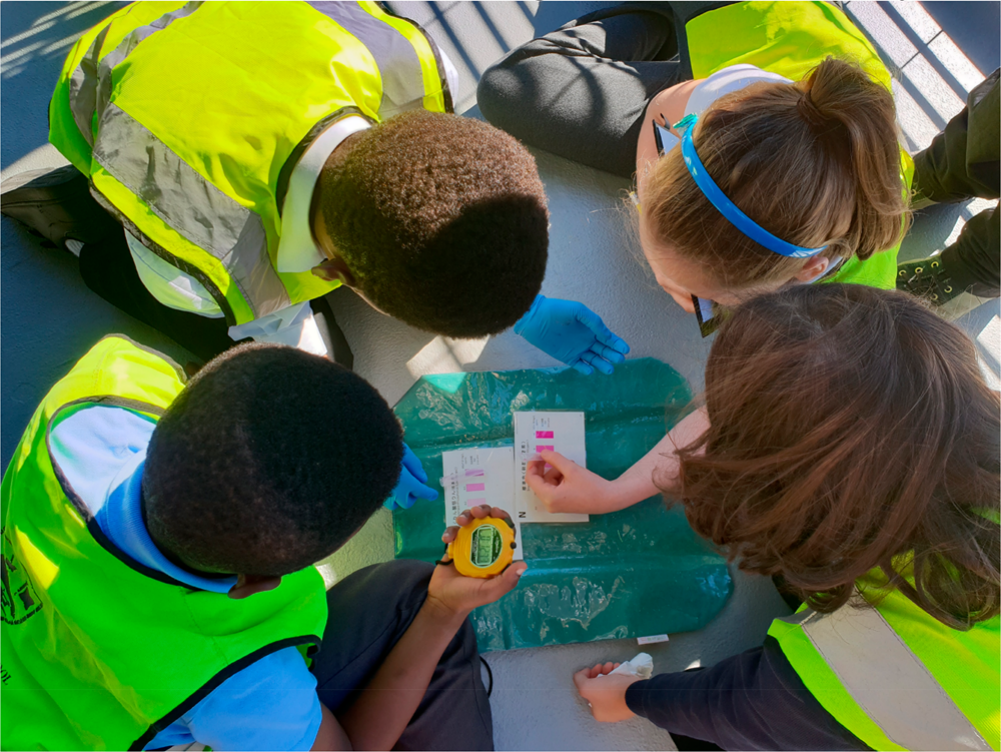
Primary (elementary) school children investigating the pH of river water.

In the U.K., science is a core subject at primary or elementary school, but the perception is that teachers, who have to teach all subjects, are not confident in teaching science and that the experience for children is rather limited. The Primary Science Teaching Trust^42^ is a charity that runs the U.K. primary science teacher of the year award annually, and around 10–20 teachers are awarded such a prize and invited to become Fellows of a virtual college. These teachers have a wide range of science qualifications—from the basic GCSE (U.K. pre-16) through to a Ph.D. in a science subject—and teach across all key stages and year groups. Therefore, some award winners have been experienced scientists, but some are not. The awardees teach children as young as 4 through 11, which is typically the oldest age at U.K. primary schools. Hence, these teachers are not “expert” scientists focused on teaching just 10–11 year olds. What makes these teachers so good? Time and time again, watching these teachers teach, some characteristics emerge. They find good contexts (see Figure 2) and are able to utilize the culture of the children in their class. They are also skilled in using the wider curriculum to incorporate scientific knowledge and understanding. An example of this would be the use of the resource 1001 Inventions, which highlights the contribution made to science by Muslim scientists in the so-called “Dark Ages” of European civilization. Anglo Saxons (a cultural group who inhabited England in the Early Middle Ages) was the context used by one teacher to introduce ideas about dyeing of cloth, the plants that were available at the time, and methods used to extract dyes. The plant-based medicines and herbs that were used for curing ailments and in cooking and the scientific methods were scaffolded and discussed in order to develop an understanding of their uses. Use of the outdoor classroom (“fieldwork” to the academic)^16^ gives different learner types an opportunity to contribute and develop. Importantly, it promotes the skill of asking questions and being able to debate and justify ideas. Such teachers are not afraid to say “I do not know, let us find out” or sometimes, “I do not know, but we will make a note of that and try and answer that question” even if they do know the answer. This holistic method of teaching encourages an immersive approach to investigations and develops critical thinking in children. In Higher Education, encouraging students to ask questions is hard; they fear asking a “silly” question or one that shows ignorance, and they expect the academic to answer any question that is posed as if they are an encyclopedia/Google. Whether deliberately or not, it is good to say “I do not know” from time to time and encourage the student to answer the question themselves. The answer the academic may give may be totally correct, but does it help the student to establish a deep understanding? The primary school setting is unique in U.K. education, in that one teacher will often teach all the children in one class for the whole year. Therefore, they have the flexibility to alter and change the material covered and the time spent on that. However, Higher Education establishments also have that freedom to include for example fieldwork, which can connect a diverse range of students with subject matter.

### Case Study 4: Service Learning as a Vehicle to Promote Inclusivity in South Africa

Over 30 years since the end of apartheid, South Africa remains one of the most unequal societies in the world. Although the government has made great strides to provide the financial resources to enable young South Africans from disadvantaged communities to enter universities, the problem of inclusivity and the general feeling of not belonging often remain. Following widespread student protests in 2015 and 2016, under the umbrella of the #Feesmustfall movement, transformation of the South African university sector to become more African relevant and more inclusive was a key student demand. A service-learning project implemented at Rhodes University in the Eastern Cape Province of South Africa^43^ provided an opportunity for learners from resource-limited schools in disadvantaged communities to engage proactively with university students and in the process begin to break down barriers to inclusivity and engender a sense of belonging.

The project, while of sound chemistry educational value, positively changed perceptions on the importance and advantages of inclusivity and diversity for those involved. Centred on a standard azo dye preparation in the undergraduate organic chemistry laboratory, undergraduate students spent the first of two laboratory sessions preparing a variety of different colored azo dyes from different starting substrates. The students compared the UV spectra of their different azo dye products and the colors produced when they dyed small pieces of cloth with their products. At the end of the laboratory session, the class reached consensus on the most attractively colored azo dye which could also be prepared in reasonable yield in the laboratory. In the service-learning component of the project that followed in the next laboratory session, the undergraduate students were subsequently graded on their efforts to guide grade 12 learners from local resource-limited schools through the preparation of this dye. The students were expected to be able to demonstrate and lead the learners through all aspects of the dye preparation from basic laboratory safety and laboratory technique to finally tie-dyeing a T-shirt with the end product.

The feedback during the laboratory session and from postlab reflection was insightful. The project not only increased the engagement of the undergraduate class with the chemistry of azo dyes but also introduced enjoyment and excitement into the chemistry laboratory in both undergraduate students and grade 12 learners (some of whom who had never been exposed to a laboratory before). A significant proportion of the undergraduate class indicated that the project had made them more aware of the inequality in South African society and the importance of their increased personal involvement in chemistry community engagement and outreach projects.^44^ They also recognized the importance of scientists engaging with the public and sharing their knowledge.^45^ One of the schoolteachers who accompanied the grade 12 learners commented after the joint laboratory session, “We always thought that the university was not for us but today we have seen that it is for us too”. Sewry and Paphitis carried out a detailed study of the project and concluded that for many undergraduate students who were not from township backgrounds, the project challenged their perceptions and beliefs about society in South Africa, it raised their social awareness and developed an enthusiasm to change and support a change in the school system, and it challenged and improved their communication skills and led to demonstrable personal growth, all while increasing their understanding of azo dyes.^44^

## Discussion

The Chemical Sciences involves some elements of laboratory work and can, as stated, be closed to groups of people, through perceptions of health and safety, ability to interact, and possibly even the arrogant notion that they would not be interested in studying these subjects. However, to progress ideas concerning the teaching of the Chemical Sciences, as diverse a group of stakeholders as possible must be engaged. Rather than considering the adaptations that may be required, we should be welcoming the different perspectives introduced and how these can advance our thinking in terms of teaching and research.

Research carried out by the Royal Society of Chemistry (RSC) in the U.K. shows that for inclusion and diversity initiatives to succeed they must also consider what helps people belong because when people feel that they belong, they are more able to share their ideas and be creative.^46^ Being told that you do not belong even indirectly (e.g., indigenous students in Australia or resource-limited students in South Africa), being the only one with a particular lived experience (e.g., visually impaired students), or being excluded by people’s assumptions, stereotypes, and biases (e.g., primary and elementary teachers) can get in the way of belonging.

From the few case studies illustrated, each cohort has contributed ideas on ways to improve teaching in the Chemical Sciences for all age groups. In the Higher Education context, where much effort is being extended to be more inclusive, engaging in more inclusive approaches will enhance learning for all. The 17 United Nation’s Sustainable Development Goals (SDGs) (https://sdgs.un.org/goals) are often held up as the ultimate aspiration for STEM endeavors, and the Chemical Sciences will need to play a central role in solving problems such as climate change, more efficient and ecofriendly agriculture and industrial processes, clean energy generation, and many more. The contributions of a diverse cohort improving and developing the way we view and teach the subject will inevitably lead to more diverse and exciting approaches to research. If we are to solve these pressing problems, we need everyone to contribute and cannot afford to exclude anyone. The Chemical Sciences need a diverse cohort to be engaged for the subject to evolve, and EDI is essential. For Chemical Sciences education across all age phases to be a truly diverse experience, we must seek to question the materials we use and find alternative examples.

## Summary and Conclusions

An increasing amount of effort is being invested into the areas of equality, diversity, and inclusion in Higher Education Institutes in general but in the Chemical Sciences community in particular. Some excellent innovations through training and laboratory innovations are moving the community in the right direction. However, beyond the obvious moral imperative to provide access to all, the immense value diverse groups bring to our understanding about how to learn and the new perspectives on how to view and solve chemical problems that they reveal are essential for the future of the subject. The four case studies demonstrate why EDI is essential and the importance of taking into consideration student’s culture, background, and abilities. Yes, these will help in the design of chemistry activities that will engage all students in learning chemistry, but it may lead to a totally new and highly effective way of engaging and learning.

## Supporting Information Available

Brief descriptions of the relevant teaching content for each of the four case studies (PDF, DOCX)
