# Supplementary material for: Flipping the Thinking on Equality, Diversity, and Inclusion. Why EDI Is Essential for the Development and Progression of the Chemical Sciences: A Case Study Approach
Source: J Chem Educ. 2023 Oct 12;100(11):4279–86. doi: 10.1021/acs.jchemed.3c00364 (PMC10653219; doi:10.1021/acs.jchemed.3c00364)
Supplement: Supplementary file 1 — ed3c00364_si_001.pdf [file ed3c00364_si_001.pdf]

SUPPORTING INFORMATION FOR THE PAPER

Flipping the thinking on equality, diversity, and inclusion, why EDI is essential for the development and progression of the chemical sciences: a case study approach.

M. Anwar H. Khan,[1] Timothy G. Harrison,[1#] Magdalena Wajrak,[2] Michele Grimshaw,[3] Kathy G. Schofield,[3] Alison J. Trew,[3] Kulvinder Johal,[3] Jeannette Morgan,[3] Karen. L. Shallcross,[1] Joyce D. Sewry,[4] Michael T. Davies-Coleman,[5] Dudley E. Shallcross[1,5*]

1. School of Chemistry, Cantock's Close, University of Bristol, Bristol, BS8 1TS, UK.
2. School of Science, 270 Joondalup Drive, Edith Cowan University, Perth, WA 6027, Australia
3. Primary Science Teaching Trust, 12 Whiteladies Road, Bristol, BS8 1PD, UK.
4. Department of Chemistry, Rhodes University, Makhanda, 6139, South Africa.
5. Department of Chemistry, Robert Sobukwe Drive, University of Western Cape, Bellville, 7535, South Africa.

Corresponding author Dudley E. Shallcross (d.e.shallcross'bris.ac.uk)

OVERVIEW

We present an overview of some of the key activities undertaken in the four case studies. This is not an exhaustive description of every activity, but sufficient description of material described in the text that would benefit from some further information.

CASE STUDIES

Case study 1: Visually impaired adults

All practical activities described went through a health and safety audit. On any modifications and subsequent approval, the practicals were conducted during office hours when additional staff were on stand-by in case of any incidents (there were none). It is noted that a special dispensation for guide dogs in the lab was granted after significant testing of the dogs response to smells in the lab. Practicals were designed not to create loud bangs and none occurred, all the dogs were well behaved and no issue occurred. However, significant planning

was required to ensure their safety and that of demonstrators. Demonstrators were aware that the dogs were present.

Summer schools ran for three consecutive years and ran from Sunday on week 1 through to Friday of week 2. The students were housed in a hall of residence and taught (non-practical) sessions were carried out in the hall of residence to reduce travel. There were twenty students on each course, and each had a sighted guide (who were trained to work with visually impaired adults but were not necessarily trained in science) for all taught (practical and non-practical) sessions. The sighted guide was assigned to the same student throughout the course. In practical sessions there three academic leads, a senior technician on hand at all times (with other technical staff being present as required depending on the practical). Five experienced demonstrators (postgraduate chemistry students who had demonstrated in the undergraduate laboratories for at least two years) were present for all practical sessions. Training from the University of Bristol disability unit was given to all staff and demonstrators before the course started.

Practical sessions were carried out in an appropriate venue, all chemistry practicals (indicated in the later schedule) were carried out in the School of Chemistry undergraduate laboratories. Any practicals involving food were conducted in a food safe laboratory, in this case guide dogs were not permitted into the venue, but students had sighted guides with them at all times.

Courses

A. What is matter made of?
B. The human body, its chemistry and structure
C. Climate chemistry

Talks associated with the course would be supported with Braille diagrams and where possible, speakers were asked to provide some tactile supporting material. Of relevance to this paper, in all three courses we ran workshops where we looked at chemical structure using moly-mod type molecular modelling kits. For courses A and C we looked at examples of gases in the air (e.g. N_2, O_2, CO_2) and for course C we looked at why some gases can be greenhouse gases. For course A and B we looked at different solid structures (crystalline and macromolecules) and where appropriate compared them with real exhibits. For course B, the students constructed amino acids and combined them into groups of 4 bonded amino acids and the students then spent time feeling the different 4-amino structures and compared this with the idea of different keys. They also constructed simple receptors for these structures and the students worked out which 4-amino structure would bind to which receptor. There was some preliminary investigation into the change of shape of the receptor as a whole on binding. We investigated polymer structures, in courses 1 and 2 initially using a workshop from school outreach called 'Why does Jelly Wobble,[S1] and then carried out practical experiments (described later) for course A and used physical models of human anatomy to distinguish between solid structures such as bone and solid structures that were flexible

such as muscle in course B. The students also had an opportunity to work with an artificial human (commonly called Stan) used by undergraduate medical students. Talks were given by members of the science, engineering and life sciences community and sessions ran for 2-3 hours and included some breaks, opportunities to explore tactile resources etc. and for students to lead discussion. The core topics covered in each course were;

Course A (core topics)

The periodic table of the elements, the building blocks of all matter.
Common gases and their structure, why is a gas a gas?
Water, the universal solvent
Liquids, their viscosity and other properties
Solids, crystalline materials, soft solids and macromolecules.
Metals, why they are good conductors but not all are magnetic (they were several other talks on metals looking at different properties)

Course B

The skeleton, its form and use and what bone is made of
Stretchy solids, why they are essential in the body and why they stretch
Inside a cell (followed by DNA workshop)
The kidney, the great shape sorter
Electrics and mechanics of the heart
The brain, the control module
The liver, the unsung hero
The circulatory system

Course C

A simple model of the Earth's climate[S2]
Ways to offset climate change[S3, S4]
The role of plants in the Earth system
Oceans and climate forcing
Air pollution and climate change
Stratospheric ozone destruction and its rebirth

The practicals undertaken during the courses were;

Titrations (to determine metals levels in water samples -linking with courses A and C and carbonate and hydrogen carbonate titrations). Students were assessed to determine what they were able to do and were comfortable to undertake in the laboratory. Some, with partial sight could set up and conduct the experiment with support. Students with no sight either had significant support to set up the experiment or had the experiment set up for them. Students (and their sighted guides) were guided through a titration and then further support was provided by demonstrators and academics as required. Some students preferred to guide the sighted guide through the experiment. The end points were determined optically, through

either a photodiode detector which the student (if they had partial sight) could detect by contrast (either colourless to coloured transition) or by the sensor being connected to an in-house talking device.

Electrochemistry[S5] (to determine electrode potentials and how these vary with concentration of solution for example) experiments linked to all courses and allowed an investigation of the concept of pH, conductivity and metal reactive series and the principles of batteries in course C in particular. Here a voltmeter was attached to a talking meter and students and their guides were able to record data and other output devices were used. In addition, in course C, we used Gratzel cells in some experiments.

Kinetics (iodination of propanone) using an optical sensor to determine the variation in iodine concentration. Experiments allowed students to determine reaction order with respect to [propanone], $[H^+]$ and [Iodine].

Perfume workshop[S6] Students were given access to nine fragrances and instructed in how to combine certain fragrances as a group. Students were then allowed to experiment with mixtures to create their own perfume.

Metal analysis
Conductivity and resistance of common metals and their alloys using appropriate meters.

Case study 2: First Peoples in Australia[S7]
A list of activities that are part of the Old Ways New Ways projects are given in table S1. We provide details of two of the activities that are particularly relevant to this paper. Typically, up to 30 students take part in any activity and these may take part indoors or outdoors.

**Activity**	**Brief description**
**1**	**Exploring the physics behind the aerodynamics of boomerangs.**
**2**	**Making aboriginal glue from charcoal, resin and kangaroo poo.**
**3**	**Traditional (modern) aboriginal glue making**
**4**	**Using ash to clean dishes (alkaline detergent)**
**5**	**Aboriginal filtering methods**
**6**	**Iodine fingerprinting and analogies with Aboriginal tracking.**
**7**	**Analysing hair types using a microscope.**
**8**	**Experimenting with the polymer polymorph.**
**9**	**Aboriginal nappies using the zamia plant and comparisons with modern nappies.**
**10**	**Odours, their chemical structure and use in Aboriginal hunting.**
**11**	**Investigating blood types.**

**12**	**Using chromatography to determine chemicals used in Aboriginal smoking ceremonies.**
**13**	**Aboriginal astronomy.**
**14**	**Aboriginal art and modern analysis.**
**15**	**Microscopical investigation of worms and plants.**

Table S1. A list of activities undertaken in the Old Ways New Ways programme.

Activity 4 Using ash to clean

Personal hygiene and cleaning of food containers and cooking utensils are key to living without illness. Aboriginal people used wood ash as a cleaning product. Combining ash with hot water forms a paste that is used to clean utensils.[S8] $CaCO_3$ is a major component of this ash and can help to dissolve fats and oils when in solution. In the investigation, comparison between modern soap and the ash in cleaning items (e.g. time taken to clean the item) is recorded and compared.

Activity 9 Zamia plant compared with modern nappies

The zamia palm is a high shrub found in the Jarrah forests in south west Western Australia. Aboriginal people used the woolly material found around the base of the fronds on top of the trunk of the Zamia as a nappy for many years before modern nappies were developed.

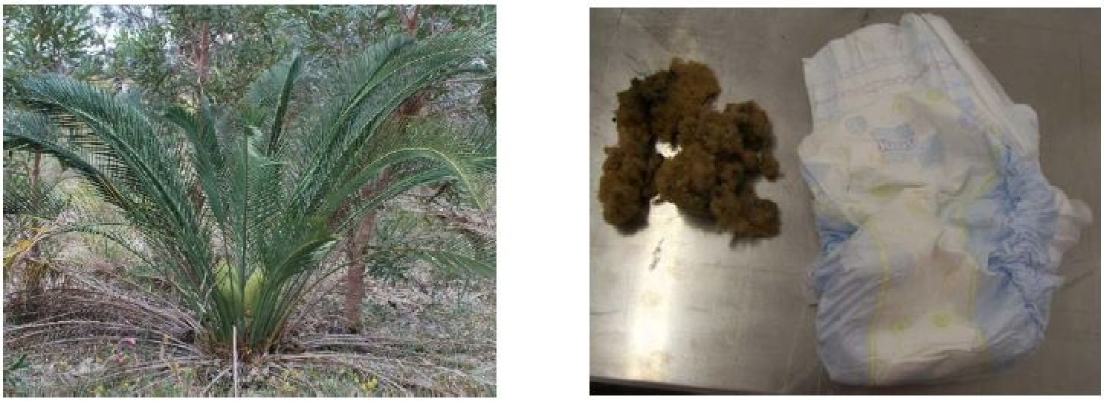

Figure S1. Zamia plant shown on the left and the woolly material from the plant on the right. Images are courtesy of the OWNW team (contact m.wajrak@ecu.edu.au).

The investigation involves comparing the absorbent lining in a modern nappy with that from the zamia plant. For a given weight of nappy and zamia plant, water is added until no more can be absorbed and the volume of water used us recorded.

Case study 3: Primary (Elementary) School Science Teachers

Relevant activities are covered in the following papers.[S9, S10]

Case study 4: Service learning as a vehicle to promote inclusivity in South Africa.[s11-13]

All the honours students who sign up for the course, participate in the service activity which takes place at two schools, with which relations have been formed over a number of years. Before participating in the service activities at the selected schools students have the opportunity to do one or two trial runs of the demonstration and experiments with an after school science club to ensure that they are ready for the course activities in the schools. For the service activity, the honours students present 'A Pollutant's Tale'[S14] and two hands-on experiments[S15] to grade 7 learners (12–14 years old) at both a well-resourced school (WRS) and an under-resourced school (URS) in the local community.

Activity	Approx time / hours
Introductory lecture	1.5
Student training on 'A Pollutant's Tale'	1.5
Check equipment	1
Practice run for 'A Pollutant's Tale'	1
Practice run for hands-on experiments	1
Activity at WRS, including travel (date and time negotiated with the school)	2
Debriefing after activity at WRS	1
Writing first reflection (2–3 pages)	1
Activity at URS, including travel (date and time negotiated with the school)	2
Debriefing after activity at URS	1
Writing second reflection (2–3 pages)	1
Writing final reflection (3–5 pages)	2

Table S2. Main activities during the course and their duration.

Summary of Assessment of the course

Social awareness

Many students did not really appreciate the daily lives of the vast majority of South African children. This program brought that onto sharp focus and raised social awareness.

Civic responsibility

Students found out the value of their interactions with students from lower economic means and why such programs are effective.

Challenging beliefs

For most students the difference between their economic status and that of the students they worked with was significant. Therefore, some student demonstrators were fearful of going to the township schools but were surprised to find that this was not the case. A key challenge was seeing that despite the vast difference in resources between state (township) schools and private schools the ability of the students within is remarkably similar.

Science communication and demonstration skills

Undertaking the demonstration lecture demanded a range of skills, preparation, timing, adapting if experiments didn't work but engaging with the audience and using appropriate language was a key response.

Personal growth

Data shows that students were challenged by the course, from communication skills to preconceived ideas about the students and reported that they had developed as a person and as a scientist.

References

S1. Shallcross, D.E.; Shallcross, N.K.R.; Schofield, K.G.; Franklin, S.D. Why does jelly wobble? A workshop for primary school children on the structure and properties of solids. *J. Emergent Science.* **2022,** *99,* 38-44.

S2. Shallcross, D.E.; Harrison, T.G. Climate Change modelling in the classroom *Science in School.* **2008**, *9*, 28-33. www.scienceinschool.org/2008/issue9/climate.

S3. Shallcross, D.E.; Harrison, T.G.; Henshaw, S.J.; Sellou, L. Looking to the heavens: Climate Change experiments. *Science in School.* **2008,** *12*, 38-43. www.scienceinschool.org/2009/issue12/climate

S4. Harrison, T.G.; Shallcross, D.E. Is climate change all gloom and doom? Introducing stabilisation wedges. *Science in School.* **2011,** *20*, 60-64.

S5. Shallcross, D.E.; Harrison, T.G. Practical demonstrations to augment climate change lessons. *Science in School.* **2008,** *10*, 46-50. www.scienceinschool.org/2008/issue10/climate

S6. Griffin, A.; Harrison T.G.; Shallcross D.E. Perfume chemistry, sexual attraction and exploding balloons: university activities for school.*Science in School.* **2006**, 3, 48-51. Available at: http://www.scienceinschool.org/2006/issue3/perfume.

S7. https://www.ase.org.uk/system/files/Old%20Ways%2C%20New%20Ways.pdf last accessed September 2023.

S8. Fontaine, R. Clean your cooking gear with wood ashes. http://survivaltopics.com/clean-your-cooking-gear-with-wood-ashes last accessed August 2023.

S9. Grimshaw, M.; Curwen, L.; Morgan, J.; Shallcross, N.K.R.; Franklin, S.D.; Shallcross, D.E. The benefits of outdoor learning on science teaching. *J. Emergent Science*, **2019,** *16,* 40-45.

S10. Grimshaw, M.; Spilsbury, N.; Sainsbury, P.; Schofield, K.G.; Tyler, P.; Shallcross, N.K.R.; Shallcross, D.E. Michele. Science days leading to Science weeks, why have them? *J. Emergent Science*, **2019,** *17,* 45-49.

S11. Glover, S.R.; Sewry, J.D.; Bromley, C. L.; Davies-Coleman, M. T.; Hlengwa, A. The implementation of a service-learning component in an organic chemistry Laboratory course. *J. Chem. Ed.* 2013, *90,* 578-583.

S12. Sewry, J.D., Paphitis, S.A. Meeting important educational goals for chemistry through service-learning. *Chem. Ed. Res. Prac.* **2018**, *19***,** 973-982**.**

S13. Sewry, J.D., Glover, S.R., Harrison, T.G.; Shallcross, D.E.; Ngcoza, K.M. Offering community engagement activities to increase Chemistry knowledge and confidence to teachers and students. *J. Chem. Ed.* **2013**, *90***,** 578-583**.**

S14. Sunassee, S.N.; Young, R.M.; Sewry, J.D.; Harrison, T.G.; Shallcross. D.E. Creating Climate Change Awareness in South African Schools Through Practical Chemistry Demonstrations. *Acta Didactica Napocensia.* **2012,** *4*, 35-48.
S15. Harrison, T.G.; Shallcross, D.E.; Norman, N.C.; Sewry, J.D.; Davies-Coleman, M.T. Publicising Chemistry in a Multi-Cultural Society: The Bristol and Rhodes Universities Chemistry Outreach Experiences. *South African Journal of Science*. **2011,** *11-12,* 1-6.
